# Effect of Circumference of Open Window Thoracostomy on Chest Wall Closure, Pleural Cavity Clearance, and Lung Expansion

**DOI:** 10.7759/cureus.18781

**Published:** 2021-10-14

**Authors:** Shagufta Nasreen, Nadir Ali, Tanveer Ahmad, Misauq Mazcuri, Ambreen Abid, Pratikshya Thapaliya

**Affiliations:** 1 Thoracic Surgery, Jinnah Postgraduate Medical Centre, Karachi, PAK

**Keywords:** bpf, open window thoracostomy, tuberculous empyema, owt, bronchopleural fistula, empyema

## Abstract

Introduction

Managing chronic empyema thoracis (CET) due to tuberculosis (TB) in debilitated patients is complicated. Open window thoracostomy (OWT) is one of the ways to manage these high-risk patients. Closure of OWT is sometimes difficult to attain. The purpose of this study is to compare the outcome of OWT in terms of chest wall closure in two similar groups. The only difference between these groups was the circumference of the OWT created. This study will benefit patients of CET with OWT to attain early chest wall closure without being subjected to another surgical trauma.

Methods

This is a prospective comparative study, conducted in the Department of Thoracic Surgery, Jinnah Postgraduate Medical Centre, Karachi, from August 2019 to July 2020. A total of 48 patients, 22 and 26 patients in group A and group B, respectively, were included in this study. Both groups were matched for age, gender, diagnosis, body mass index, and stage of empyema, with the difference only in the OWT circumference.

Results

Both groups had a history of multiple chest tube intubations. Among group A patients, a smaller circumference of OWT (20-24 cm; mean 22 cm) was created as compared to group B (30-34 cm; mean 33 cm). Spontaneous OWT closure was seen in 21 (95.5%) patients in group A and seven (26.9%) patients in group B in a time period of 6.2 ± 1.5 and 11.4 ± 0.5 months, respectively (p-value: ≤ 0.001). Pleural cavity clearance was attained in 21 (95.5%) patients in group A and 24 (92.35%) patients in group B in a time duration of 4 ± 1.4 months and 4 ± 4.1 months, respectively (p-value: ≤ 0.97). Complete lung expansion was found in 21 (95.5%) patients in group A and 24 (92.3%) patients in group B in a time duration of 5 ± 1.7 months and 4.7 ± 1.6 months, respectively (p-value: ≤ 0.62).

Conclusion

This prospective single-center study shows that successful spontaneous early closure of OWT primarily depends on the size of the OWT created. A smaller-sized OWT, if created judiciously, not only closes spontaneously but also facilitates the clearance of purulent discharge and potentially helps in the definitive healing of bronchopleural fistulae and consequent lung expansion, thereby avoiding more invasive procedures like decortication in a debilitated patient. Furthermore, there is no need for a second surgery for closure of OWT.

## Introduction

Empyema thoracis is defined as the presence of pus in the pleural space with lung entrapment. It is divided into three stages: stage I is an acute exudative phase of one to two weeks; stage II is a subacute fibro-purulent phase of two to four weeks, and stage III is the chronic organizing phase or chronic empyema thoracis (CET) of four or more weeks [1]. Of all causes of CET, tuberculosis (TB) remains the most common cause in developing countries, causing significant morbidity and mortality^ ^[2]. TB is one of the major public health problems in Pakistan, with the country ranking fifth among TB high-burden countries worldwide. Estimated TB incidence and prevalence is 267/100000 and 341/100000, respectively, with an estimated 525,000 new cases each year. Furthermore, 5-10% of people with latent TB infection will have active TB over the course of their lives. The risk of occurrence of active TB depends on several factors, including low immunity, diabetes mellitus, human immunodeficiency virus (HIV) infection, and low body mass index (BMI) associated with inadequate nutrition [3-6].

Stage I and II respond well to conservative management, including aspiration thoracocentesis, radiologically guided catheter drainage, and tube thoracostomy [7]. Stage III empyema (CET), however, requires major surgical approaches including decortication via open thoracotomy, video-assisted thoracoscopic (VATS) surgery, rib resection, and open drainage with or without muscle flap [8-9]. Most TB patients, who are chronically ill, are nutritionally deficient and debilitated. In these patients, tolerance to major surgical procedures is poor and VATS is technically not possible because of contracted thoracic cavity or fibrothorax. It is recommended to perform open drainage via open window thoracostomy (OWT) in these patients as opposed to decortication [10-11].

OWT is a less invasive surgical technique for drainage of the pleural cavity in these debilitated patients[7]. Open drainage was first described by Samuel Robinson for non-tuberculous empyema. This technique was adopted by Leo Eloesser for TB empyema in 1935 and later modified by Symbas and colleagues. This technique is helpful in clearing pus from the cavity, and subsequent lung expansion[7]. Although OWT is a life-saving procedure in these patients, the chest wall may take years to close and may require a second surgery for chest wall closure [10]. Several techniques have been proposed to facilitate early chest wall closure, including multiple debridements under general anesthesia, followed by chest wall closure in single hospital admission, second surgery for chest wall closure after clearance of pleural cavity from pus, and vacuum-assisted chest wall closure, which requires prolonged hospital stay and cost.

In this study, we will observe spontaneous chest wall closure in two different circumferences of OWT. The aim of this study is to prospectively identify factors promoting successful, spontaneous closure of the chest wall along with clearance of pleural cavity and lung expansion with minimal surgical discomfort.

## Materials and methods

This is a prospective comparative study, conducted in the Department of Thoracic Surgery, Jinnah Postgraduate Medical Centre (JPMC), Karachi, from August 2019 to July 2020. Informed consent was obtained from patients and the study was approved by the Institutional Review Board (IRB no.: F.2-81/2019-GENL/30036/JPMC, dated July 27, 2019). We recruited 48 patients for this study. Patients were randomized into two groups via online randomization software, Research randomizer (https://www.randomizer.org/), i.e. 22 and 26 patients in group A and group B, respectively. Both groups were matched for age, gender, diagnosis, body mass index (BMI), and stage of empyema. The only difference was in OWT circumference. The circumference of OWT in group A was in the range of 20-24 cm (mean: 22 cm) while group B had a circumference of 30-34 cm (mean: 33 cm).

Cachectic patients with unilateral radiologically diseased lung, and BMI <20 kg/m^2^ who are not fit for major surgery and failed multiple tube thoracostomies, with the persistence of empyema, were included in the study. Patients with CET secondary to causes other than TB and those who were fit for pulmonary decortication were excluded. All patients were referred from hospital pulmonologists with a diagnosis of CET secondary to pulmonary, extrapulmonary, or reactivation of TB (Table 1).

**Table 1:** Diagnosis of tuberculosis AFB: acid fast bacilli

Diagnosis	Group A (n=22)	Group B (n=26)
Sputum for AFB smear/gene Xpert	2	4
Pleural fluid for AFB smear/gene expert	4	3
Radiological features	10	11
Defaulter with radiological signs	6	8

Chest X-ray and BMI were done on admission, first postoperative day, fortnightly for one month, and then monthly for the rest of the year. In the postoperative period, anti-tuberculous therapy (ATT) was continued and empiric antibiotic was given for seven days to those who did not show any growth on culture; otherwise, antibiotics were prescribed according to culture and sensitivity.

Operative technique

Patients were operated under general anesthesia in a double-lumen endotracheal tube in a standard thoracotomy position. To protect the airway from aspiration, the collection was drained through the previous thoracostomy site in the supine position. The incision was made on the lateral chest wall in the most dependent part after confirming the position of the diaphragm on chest X-ray and needle aspiration of pus or air. A segment of one or two ribs was excised depending on fibrosis of the thoracic cavity and crowding of ribs to make the desired OWT size (Group A or B). The empyema cavity was entered after the pleura was incised. A biopsy of pleura and a sample of purulent material was obtained. The loculations were manually broken down and cavity washed with warm saline. If a bronchopleural fistula (BPF) was present and within reach, it was repaired with 2/0 prolene. The skin edges of OWT were sutured with chest wall muscles and pleura with vicyrl 0 in an interrupted manner. No flap was created and OWT was kept open. Hemostasis was secured and the wound packed with gauze.

Nursing care was taught to all patients and their attendants. This included lying on the operated side, change of dressing when soaked, daily washing of pleural space with normal saline, incentive spirometry, and climbing stairs. Diet charts were given to every patient, mentioning carbohydrate, protein, and fat ingredients to attain their normal BMI. Cavity clearance and OWT closure were noted clinically by gross examination and wound contraction. Lung expansion was observed on serial chest X-rays.

Statistical analysis

Data were entered, analyzed, and processed through Statistical Package for the Social Sciences (SPSS), version 22 (IBM Corp., Armonk, NY). Qualitative variables like gender, previous chest intubation, lung expansion, treatment compliance, and co-morbidities were presented as frequency and percentages. Quantitative variables like age, weight, BMI, time for lung expansion, and chest wall closure time were presented as mean and standard deviation. Data were compared in both groups using the chi-square test for qualitative variables and the independent t-test for quantitative variables. A p-value of ≤0.05 was taken as significant. Data are presented in tabulated form.

## Results

A total of 48 patients with a history of TB CET were included in this study. Patients were randomized into groups A and B with 22 and 26 patients, respectively, on the basis of the circumference of OWT. The mean age of group A patients was 31.59 ± 12.9 years and in group B, it was 29.23 ± 10.6 years. There were 16 males and six females in group A, and 17 males and nine females in group B. Both groups had a history of multiple chest tube intubations. Among group A patients, a smaller circumference of OWT (20-24 cm; mean: 22 cm) was created as compared to group B (30-34 cm; mean: 33 cm) (p-value: ≤0.41). All demographic and clinical details of patients were recorded in pre-devised proforma after their consent (Table 2).

**Table 2:** Patients demographics ATT: antituberculous therapy, BMI: body mass index, BPF: bronchopleural fistula, kg/m^2^: kilograms per square meters, SD: standard deviation

Characteristics		Group A	Group B	p-value
Age (years)	Mean ± SD	31.59 ± 12.9 (13-50)	29.23 ± 10.6 (14-55)	0.49
BMI (kg/m2)	Mean ± SD	16.75 ± 2.34 (12.5-19.9)	16.65 ± 2.17 (12.5-19.5)	0.87
Gender	Male	16	17	0.58
	Female	6	9	
BPF	Yes	9	6	
	No	13	20	
Previous tubes	1	5	3	0.58
	2	10	14	
	3	7	9	
Compliant to ATT	Yes	21	24	0.65
	No	1	2	

Spontaneous OWT closure, pleural cavity clearance (PCC), and lung expansion (LE) were comparable in both groups. Spontaneous OWT closure was seen in 21 (95.5%) patients in group A and seven (26.9%) patients in group B in a time period of 6.2±1.5 and 11.4±0.5 months, respectively. Pleural cavity clearance (PCC) and lung expansion (LE) were observed in both groups. PCC was attained in 21 (95.5%) patients in group A and 24 (92.35%) patients in group B in a time duration of 4±1.4 and 4±1.1 months, respectively. Complete LE was found in 21 (95.5%) patients in group A and 24 (92.3%) patients in group B, in a time duration of 5±1.7 and 4.7±1.6 months, respectively. When OWT closure time was compared between both groups, it was found that OWT closed earlier in group A (p ≤ 0.001), however, PCC and LE of both groups occurred in almost similar time duration with p ≤ 0.97 and p ≤ 0.62, respectively (Table 3).

**Table 3:** Comparison of both groups in terms of OWT closure, PCC, and LE LE: lung expansion, OWT: open window thoracostomy, PCC: pleural cavity clearance

Characteristics	Group A	Group B	p-value
Spontaneous OWT closure	21 (95.5%)	7 (26.9%)	0.001
Time to OWT closure (months)	4-9 (6.28 ± 1.55)	11- beyond 12 (11.42 ± 0.53)	0.001
PCC	21 (95.5%)	24 (92.35%)	0.65
Time for PCC (months)	2.5-7 (4.09 ± 1.41)	2-6 (4.08 ± 1.17)	0.97
LE	21 (95.5%)	24 (92.3%)	0.65
Time for LE (months)	4-8 (5.00 ± 1.70)	4-7 (4.79 ±1.60)	0.62

BPF was present in 15 patients. It was repaired in four patients of group A and two patients of group B while the rest healed spontaneously after the creation of OWT and control of pleural infection with ATT. In group A, only one (4.5%) patient failed to close OWT. This patient was noncompliant to ATT, also failed to gain weight, and had incomplete PCC and LE. One patient with positive HIV status, compliant to ATT, gained 4 kgs of weight in eight months duration, showed PCC in three months, full LE in four months, and OWT closure in eight months. Another female patient with a known case of hepatitis C, compliant to ATT, gained 3 kgs of weight in six months; showed PCC in two months, full LE in six months, and OWT closure in seven months. It shows in group A patients despite having comorbidity and minimal weight gain attained OWT closure with success. In group B, out of 26 patients, 19 (73.07%) failed to close OWT despite all of them gained PCC and LE. Only two patients did not show PCC and LE, both were noncompliant to ATT. Improvement in BMI was observed in both groups. However despite gaining weight and improved nutrition; OWT closure was not appreciable in group B patients due to the larger circumference of OWT. The complications of both groups are shown in Table 4.

**Table 4:** Complications of OWT in Group A and Group B OWT: open window thoracostomy

Groups	Non-expanding lung	Incomplete cavity clearance	Non-closure of the chest wall	Hemorrhage	p-value
Group A (n=22)	1 (4.5%)	1 (4.5%)	1 (4.5%)	0 (0%)	0.65
Group B (n=26)	2 (7.69%)	2 (7.69%)	19 (73.07%)	0 (0%)	

**Figure 1:**
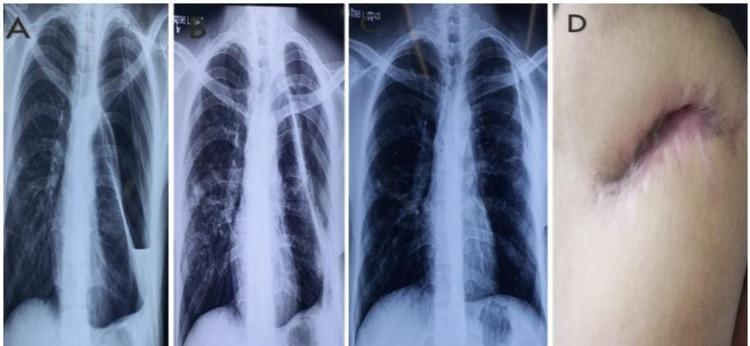
Serial chest X-rays of a patient in Group A showing progressive lung expansion and OWT closure A: Preoperative chest X-ray with air-fluid level and entrapped lung; B: Postoperative chest X-ray after 15 days showing resolution of fluid level and lung expanded apically; C: Postoperative chest X-ray at fifth month with complete lung expansion; D: OWT closure at 6th month OWT: open window thoracostomy

**Figure 2:**
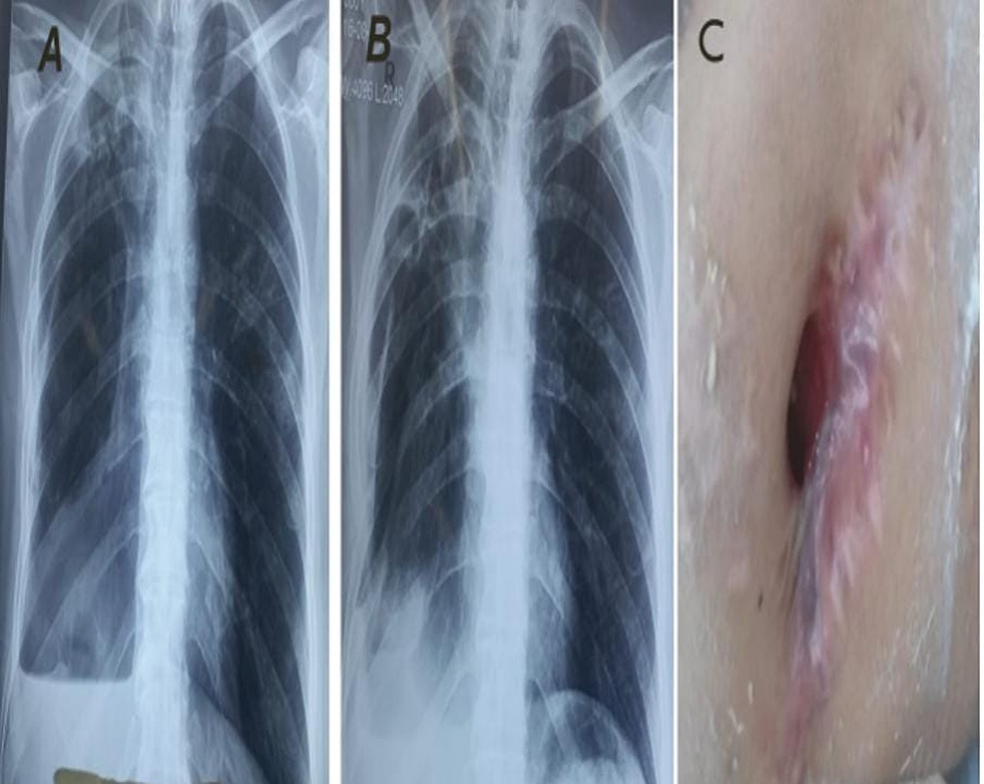
Serial chest X-rays of a patient in Group B showing progressive lung expansion, with non-closure of OWT A: Preoperative chest X-ray with air-fluid level; B: Postoperative chest X-ray at 4th month with complete lung expansion; C: Postoperative 11th month with chest wall showing non-closure of OWT OWT: open window thoracostomy

Figure 1 shows the overall success rate of Group A patients (Figure 1) as compared to Group B patients (Figure 2), as they got PCC and LE along with OWT closure.

## Discussion

The diagnosis of CET is based on history, clinical signs and symptoms, along with microscopic and radiological features. As soon as the diagnosis of CET is confirmed, an adequate drainage procedure should be instituted. The management of CET depends on surgical principles proposed by Graham; drainage of empyema cavity and obliteration of pleural space [7].

Definitive treatment of choice for CET depends on the patient’s general condition based on nutritional status, condition of the underlying lung, cause of empyema, presence or absence of bronchopleural fistula (BPF), and duration of ATT taken. Managing patients with tubercular empyema (TB empyema) is complicated due to late presentation, chronicity of disease, along with noncompliance to ATT. In debilitated patients with CET, major surgical procedure is poorly tolerated, and it is recommended to perform procedures with minimal surgical discomforts like VATS and open window thoracostomy (OWT) [10-11].

VATS although associated with minimal surgical discomfort, is efficient till stage II empyema [11-12]. However, the presentation of most patients is in the third stage (organized) with a narrow, twisting thoracic cavity and rib crowding which is not favorable for thoracoscopic decortication nor sufficient [11].

OWT allows egress of purulent fluid from pleural space, thinning of cortical peel, and eventual expansion of the lung to fill the thoracic space. In 1935 Leo Eloesser performed OWT for TB empyema and this was later modified by Symbas and colleagues in 1971 [13]. OWT is a lifesaving procedure in cachectic patients with CET.

Leo Eloesser performed OWT for TB empyema after resecting a portion of the ribs and creating an open wound with a muscle flap in the most dependent part of the chest. This technique is helpful in clearing pus from the cavity and subsequent LE. The presumed purpose of constructing a flap was to create a one-way valve that allows the egress of pus and prevents air from entering the thoracic cavity [13]. Open drainage is an ancient technique for empyema management and was created as a permanent open wound, which is then closed secondarily by muscle flap, antibiotics, or simple chest wall closure [11-12]. Clagett also described the method of open drainage in a three-staged procedure for empyema thoracis: open drainage, serial debridements, and chest wall closure [12]. Eloesser’s method of open drainage is permanent, with the wound open throughout a patient’s life.

OWT closure is important to improve the patient’s quality of life. The major complication of this procedure is non-closure of the chest wall for several years or the need for a second surgery for chest wall closure. Several authors suggested second surgery to obliterate pleural space and chest wall closure [10].

Our study revealed that spontaneous OWT closure is possible within months; this depends on the surgical technique and wound circumference. Smaller wounds close earlier as compared to larger ones, with the same results in terms of PCC and LE. We have changed the surgical technique of open drainage from Eloesser’s. We believe that in the organized phase of empyema in adults, the mediastinum is fixed due to repeated previous infections, and creating a one-way valve will not add any benefit to the patient’s treatment. We modified the OWT technique by not forming a muscle flap and control of hemostasis was secured by suturing of skin with chest wall muscles and pleura. This technique promotes progressive contraction of the wound over time, and the wound closes spontaneously with enough time for PCC and LE.

Several authors have studied open drainage methods, outcomes, and closure techniques. OWT closure can be attempted with muscle flap transposition, antibiotics, or simple chest wall closure when the patient’s condition is satisfactory for a second surgery. Most authors agreed on the significance of OWT closure to improve patient’s life.

Tai Hato et al. retrospectively studied 35 patients with heterogeneous causes of CET, who had undergone OWT. The second surgery was attempted in 12 patients after a median time of 4.5 months (2.50-41.60) for OWT closure. Complete closure was achieved in 10 (34%) patients only out of 35, whereas in our study, we achieved a 95% closure rate in group A. Tai Hato worked to investigate predictors of successful OWT closure and concluded nutrition as an important factor for OWT closure. This finding is consistent with our experience based on BMI improvement, in which nutrition is an important but not the only factor for OWT closure [14]. BMI improvement and OWT closure among group A patients were statistically significant owing to the fact that nutrition played a role in OWT closure.

A study by Massera et al. on OWT for empyema reported a 90% window closure rate with a second surgery (median time 5 months). The cavity was obliterated with muscle and antibiotics [10]. Reyes et al. reported only 22% of his patients gained closure of OWT after the second surgery with a median time of 454 days (90 days - 3 years) [15].

In the past few years, vacuum-assisted closure (VAC) of the empyema cavity was introduced, claiming rapid healing and chest wall closure. Palmen et al. compared OWT with and without the VAC technique and reported early eradication of pus from the pleural space and re-expansion of pulmonary parenchyma in patients treated with VAC [16]. Although OWT with VAC showed good results, it is expensive, along with requiring serial debridement under general anesthesia. In our study, conventional OWT showed the same results for PCC and LE; additionally, the chest wall was spontaneously closed without needing second surgery or prolonging the hospital stay.

Sziklavari et al. showed the VAC of OWT and reported early OWT closure in a single admission, but the study contained 87% cases of stage II empyema and only 13% had stage III empyema [17]. All his patients required multiple debridements under general anesthesia along with the change of dressing twice weekly, resulting in a long hospital stay and increasing cost. Although the success rate was higher in terms of control of infection and OWT closure, all patients needed a second surgery for OWT closure.

In comparison to other studies, none of our patients required a second surgery for OWT closure. Pleural cavity clearance with simultaneous lung expansion along with spontaneous OWT closure was attained in 95% of cases of Group A patients. Adaptation of this surgical technique without flap or vacuum along with creating a smaller circumference of OWT results in spontaneous chest wall closure. Results of Groups A and B for PCC and LE were almost similar. It means that a larger circumference of OWT is not a requirement for effective PCC and LE. A smaller circumference will not only benefit patients in terms of chest wall closure but also be effective in complete LE and PCC.

In our opinion, dressings should be changed twice or thrice daily depending on soakage along with washing the cavity with saline. There is no need for serial debridement under local or general anesthesia. Patients are taught to lie on the operated side for the maximum duration, to facilitate the drainage of pus from the cavity. Egress of pus combined with a good diet, ATT, physical activity, and incentive spirometry helps control sepsis and, in turn, re-expansion of lung parenchyma as the cavity gets rid of purulent collection.

Although compliance with ATT is required for PCC and LE, early OWT closure is mainly dependent on OWT circumference.

Thourani et al., in their 26 years of experience with a modified Eloesser flap, reported a mean length of stay (LOS) in a hospital of 16 days. Sziklavari et al. reported a LOS of 21 days (median 6 to 51 days). In our study, LOS in the hospital was four days (range 3 to 6) for both groups, which is the shortest to date and without postoperative complications in hospital and at home.

**Table 5:** Comparison of outcome of OWT in debilitated patients by different authors OWT: open window thoracostomy, VAC: vacuum-assisted closure

Author	Number of patients	Cause of empyema	Spontaneous closure	Attempted closure	Duration	Surgical technique	Successful closure
Thourani et al. [7]	78	Heterogeneous	Permanent window	None		Eloesser	Permanent window
Massera et al. [10]	19	Heterogeneous	0	10	5 months (3-9)	Eloesser	9 (52%)
Hato et al. [14]	35	Heterogenous	0	12	4.5 months	OWT	12 ( 34%)
Reyes et al. [15]	78	Heterogenous	2	15	5 months	Eloesser	17 (22%)
Sziklavari et al. [17]	43	Heterogeneous	0	34	5-48 days	OWT-VAC	33 (76.7%)

This is a single-center study that only included tuberculous CET patients, excluding other causes of empyema. Since the study was conducted in a single institute, the sample size was less diverse and limited. Therefore, more studies, including large and diverse sample sizes, should be conducted.

## Conclusions

This was a prospective comparative single-center study, which encompassed debilitated patients with empyema thoracis who could not tolerate major surgical intervention and benefited with OWT. OWT facilitates the clearance of pus from the pleural cavity and lung expansion but leaves the patient with complications of non-closure of the chest wall. We compared a larger circumference OWT with a smaller circumference OWT and concluded that a smaller circumference OWT will not only be beneficial in the clearance of the pleural cavity and lung expansion but will also help in spontaneous successful closure of the chest wall without the need for a second surgery. Creating a circumference of 20-24 cm for OWT in the lateral chest wall is efficient for effective pleural cavity clearance and spontaneous chest wall closure. Thus patients can more readily return to normal routine life.
